# A strategic study of acupuncture for diabetic kidney disease based on meta-analysis and data mining

**DOI:** 10.3389/fendo.2024.1273265

**Published:** 2024-02-26

**Authors:** Yunfeng Yu, Gang Hu, Xinyu Yang, Yuman Yin, Keke Tong, Rong Yu

**Affiliations:** ^1^ Department of Endocrinology, The First Hospital of Hunan University of Chinese Medicine, Changsha, Hunan, China; ^2^ College of Chinese Medicine, Hunan University of Chinese Medicine, Changsha, Hunan, China; ^3^ Department of Gastroenterology, The Hospital of Hunan University of Traditional Chinese Medicine, Changde, Hunan, China

**Keywords:** diabetic kidney disease, meta-analysis, data mining, acupuncture, acupoint

## Abstract

**Objective:**

The specific benefit and selection of acupoints in acupuncture for diabetic kidney disease (DKD) remains controversial. This study aims to explore the specific benefits and acupoints selection of acupuncture for DKD through meta-analysis and data mining.

**Methods:**

Clinical trials of acupuncture for DKD were searched in eight common databases. Meta-analysis was used to evaluate its efficacy and safety, and data mining was used to explore its acupoints selection.

**Results:**

Meta-analysis displayed that compared with the conventional drug group, the combined acupuncture group significantly increased the clinical effective rate (risk ratio [RR] 1.35, 95% confidence interval [CI] 1.20 to 1.51, P < 0.00001) and high-density lipoprotein cholesterol (mean difference [MD] 0.36, 95% CI 0.27 to 0.46, P < 0.00001), significantly reduced the urinary albumin (MD –0.39, 95% CI –0.42 to –0.36, P < 0.00001), urinary microalbumin (MD –32.63, 95% CI –42.47 to –22.79, P < 0.00001), urine β2-microglobulin (MD –0.45, 95% CI –0.66 to –0.24, P < 0.0001), serum creatinine (MD –15.36, 95% CI –21.69 to –9.03, P < 0.00001), glycated hemoglobin A1c (MD –0.69, 95% CI –1.18 to –0.19, P = 0.006), fasting blood glucose (MD –0.86, 95% CI –0.90 to –0.82, P < 0.00001), 2h postprandial plasma glucose (MD –0.87, 95% CI –0.92 to –0.82, P < 0.00001), total cholesterol (MD –1.23, 95% CI –2.05 to –0.40, P = 0.003), triglyceride (MD –0.69, 95% CI –1.23 to –0.15, P = 0.01), while adverse events were comparable. Data mining revealed that CV12, SP8, SP10, ST36, SP6, BL20, BL23, and SP9 were the core acupoints for DKD treated by acupuncture.

**Conclusion:**

Acupuncture improved clinical symptoms, renal function indices such as uALB, umALB, uβ2-MG, and SCR, as well as blood glucose and blood lipid in patients with DKD, and has a favorable safety profile. CV12, SP8, SP10, ST36, SP6, BL20, BL23, and SP9 are the core acupoints for acupuncture in DKD, and this program is expected to become a supplementary treatment for DKD.

## 1 Introduction

Diabetic kidney disease (DKD) is a metabolic disease characterized by systemic metabolic disturbance (1) and is the most common and severe complication of diabetes (2). DKD manifests as hyperfiltration and proteinuria in the early stage and end-stage renal disease in the late stage (3). Epidemiological studies have shown that the number of patients with DKD is increasing year by year, and it is estimated that there will be 592 million patients with DKD by 2035, accounting for about 8%–10% of the global population (4, 5). Renal failure in patients with advanced DKD often requires renal replacement therapy or renal transplantation, which seriously jeopardizes the physical and mental health of patients and imposes a substantial economic burden on families and society (6). Currently, the main therapeutic options for DKD include hypoglycemia, antihypertensive, regulation of lipid metabolism disorders, reduction of urinary protein, and renal replacement therapy for end-stage renal disease (7). Although these treatment regimens reduce complications, delay the progression of DKD, and improve long-term survival, it remains unsatisfactory (8). Therefore, it is essential to explore a safe and effective treatment that can improve the prognosis of DKD.

Acupuncture is a traditional Chinese medicine practice that works by stimulating specific sites (acupoints) on the surface of the body (9) and is a widely accepted complementary and alternative therapy (10). Previous studies have indicated that acupuncture can attenuate insulin resistance in patients with type 2 diabetes, implying that it may have the potential to improve the prognosis of type 2 diabetes and its complications (11). In recent years, more and more studies have demonstrated the unique advantages of acupuncture combined with conventional medications in treating DKD (12). Acupuncture has been reported to reduce proteinuria, glycemia, and lipids and improve patients’ quality of life with DKD (13). However, the specific benefits and acupoints selection of acupuncture for DKD is still controversial. Therefore, this study used meta-analysis to assess the efficacy and safety of acupuncture in DKD to explore its specific benefits and risks and used data mining to explore the core acupuncture points for acupuncture in DKD.

## 2 Methods

### 2.1 Meta-analysis methods

This study strictly followed the Preferred Reporting Items for Systematic reviews and Meta-Analyses (PRISMA) (14).

#### 2.1.1 Literature search

China National Knowledge Infrastructure Database (CNKI), China Biomedical Literature, China Science and Technology Journal Database, Wanfang Database, Embase, PubMed, the Cochrane Library, and Web of Science were searched for clinical studies on acupuncture for DKD up to June 2023, with subject terms covering acupuncture and DKD. Based on the subject words, the free words were expanded with the help of CNKI and Mesh databases, and then the subject words and free words were combined for retrieval. The English search formula was: ([“acupuncture” OR “acupressure” OR “needling” OR “needle therapy” OR “meridian therapy”] AND [“diabetic kidney disease” OR “diabetic kidney diseases” OR “diabetic nephropathies” OR “diabetic nephropathy” OR “diabetic glomerulosclerosis” OR “diabetic renal disease” OR “diabetic kidney injury” OR “diabetic renal injury”]). The Chinese search formula is: ([“针刺” OR “针灸” OR “针法”] AND [“糖尿病肾病” OR “糖尿病性肾病” OR “糖尿病肾小球硬化症” OR “糖尿病性肾小球硬化症”]).

#### 2.1.2 Inclusion and exclusion criteria

Inclusion criteria: (1) Randomized controlled trials; (2) The subjects included were patients with DKD (15); (3) Patients in the control group received conventional treatment, and patients in the experimental group received acupuncture and conventional treatment; (4) Efficacy endpoints included clinical effective rate, renal function (urinary albumin [uALB], urinary microalbumin [umALB], urine β2 microglobulin [uβ2-MG], serum creatinine [SCR]), blood glucose (glycated hemoglobin A1c [HbA1c], fasting blood glucose [FBG], 2h postprandial plasma glucose [2h PPG]), blood lipid (total cholesterol [TC], triglyceride [TG], high-density lipoprotein cholesterol [HDL-C]). The clinical effective rate was defined as the percentage of symptoms and signs relieved out of the total number. Safety endpoints were adverse events.

Exclusion criteria: (1) The literature had been published repeatedly; (2) The data was incomplete; (3) The data was not available.

#### 2.1.3 Literature screening, data statistics and risk of bias

First, the basic literature was imported into Reference Aid for Medicine, and the included literature was obtained by screening layer by layer according to the inclusion and exclusion criteria. Second, the included literature was sorted out, and the baseline data of each study was entered into the basic characteristics table. Furthermore, the risk of bias was assessed using the Cochrane risk of bias assessment tool. These works were completed independently by Yunfeng Yu and Xinyu Yang, and any objection was decided by Keke Tong.

#### 2.1.4 Statistical analysis

Meta-analysis was performed using Revman 5.3, with risk ratio (RR) as effect sizes for dichotomous variables and mean difference (MD) for continuous variables. Heterogeneity analysis was conducted by I^2^ test. Fixed-effects models were used when I^2^ < 50%, and the methodological quality and clinical design of the included studies were similar. A random-effects model was used when I^2^ ≥ 50% or there was significant methodologic heterogeneity or clinical heterogeneity. Leave-one-out sensitivity analysis was used to assess the robustness of the combined results and to check for the presence of individual studies that significantly affected the results. Subgroup sensitivity analysis was used to assess the effects of gender, age, and number of acupuncture points on the combined results. Stata15.0 was used to perform Harbord regression to evaluate publication bias, and if P > 0.1, it indicated no publication bias.

### 2.2 Data mining methods

#### 2.2.1 Literature search

The literature search strategy was the same as the meta-analysis part.

#### 2.2.2 Inclusion and exclusion criteria

Inclusion criteria:

(1) Randomized controlled trials or case-control studies; (2) The included subjects were patients with DKD; (3) The experimental group was treated with acupuncture, and the acupoint records were complete; (4) The efficacy of acupuncture was definite.

Exclusion criteria:

(1) Duplicate publication; (2) Data were not available; (3) External treatments other than acupuncture were used.

#### 2.2.3 Standardization of acupoint names

The names of acupoints included in the studies were standardized based on the “Nomenclature and location of meridian points” (16). For example, “Yishu” was standardized as “EX-B3”, and “Zhongjixue” was standardized as “CV3”.

#### 2.2.4 Data analysis

First, the Traditional Chinese Medicine Case Cloud V2.3 was utilized to conduct frequency analysis, with a frequency threshold set at >20% to identify common acupoints for treating DKD through acupuncture. Second, the identified common acupoints were inputted into SPSS Modeler 18.0 for association rule analysis. The analysis employed the Apriori model with parameters set at support ≥ 30%, confidence ≥ 100%, and lift ≥ 1.0. Support measured the frequency of an itemset appearing in the dataset. Confidence represented the likelihood of another subsequent itemset occurring given a set of prerequisite items. Lift was used to determine whether a rule has practical application value. Based on this parameter, the association rule analysis could obtain core acupoint combinations with practical significance consisting of acupoints with a high frequency of occurrence and close connection with each other. The acupoints that make up the core acupoint combinations were defined as core acupoints. Third, the core acupoints were imported into SPSS Statistics 25.0 for factor analysis. The factor analysis, employing principal component analysis and the rotation maximum variance method, aimed to clarify the grouping of these core acupuncture points. Factor analysis was a commonly utilized technique for reducing data dimensions, aiding in the discovery of the latent structures behind observed variables. Furthermore, factor analysis based on principal component analysis could transform multiple correlated observed variables into a few independent principal components, thereby simplifying the data structure and uncovering the inherent connections between variables. It contributed to understanding how core acupoints are grouped and subsequently analyzing the efficacy of different acupoint groups in treating DKD.

## 3 Results

### 3.1 Meta-analysis results

#### 3.1.1 Literature screening

**Figure 1:**
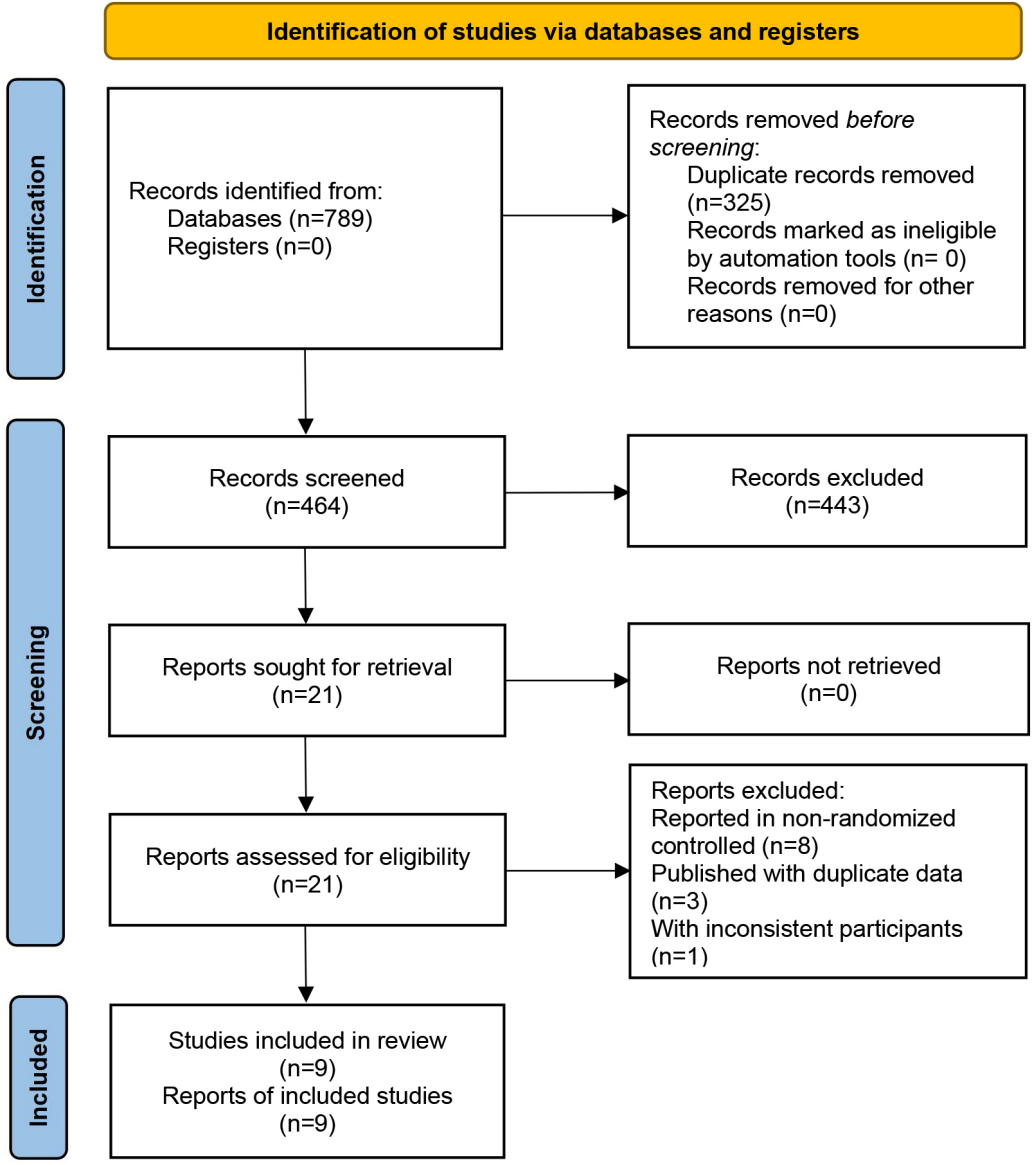
Literature screening process.

A total of 789 relevant studies were obtained, and 325 were excluded due to duplication or other reasons. After reading the title and abstract, 443 studies were eliminated. After reading the complete text, 12 studies were removed. Nine clinical studies were finally included, as shown in **Figure 1**.

#### 3.1.2 Basic characteristics of included studies

A total of nine clinical studies (17–25) (all study centers were in China) were included with a total sample size of 659 cases, of which 270 cases received conventional treatment and 389 cases received acupuncture combined with conventional treatment. Baseline information such as gender, age, and disease duration were comparable between the experimental and control groups in each included study. The basic characteristics of the included studies are listed in **Table 1**.

**Table 1:** Basic characteristics of the included studies.

Author Name	Research Center	Sample Size (E/C)	Age (Years)	Male (%)	Disease Duration (years)	Mogensen Stages	Conventional Treatment	Acupuncture Treatment	Acupoints	Treatment Duration (weeks)
Chen GC 2006 (17)	China	30/30	55.5	56.7	10.5	III 61.7% IV 38.3%	Insulin 14–60u/d α-glucosidase inhibitors 50mg tid Fosinopril 10mg bid Atorvastatin 10mg qd Dipyridamole 50mg tid	Moderate amount of time, once a day	BL23, KI3, SP6	8
Chu Q 2003 (18)	China	24/30	62.6	44.4	2.0	III 53.7% IV 46.3%	Gliquidone 30–120mg Qd Captopril 12.5–50mg Qd	20 minutes each time, once a day	BL23, BL20, BL18,EX-B3, CV4, KI3, ST36, SP9, SP6	4
Fan C 2011 (19)	China	25/28	62.0	45.3	2.0	III 47.2% IV 52.8%	Acarbose 50–100mg Tid	35 minutes each time, once every two days	BL23, BL27, BL22, BL29, BL49, BL21, KI2, LR2, KI3, ST36, TE4, TE1, LI11, CV24, CV4	6
Ji XQ 2004 (20)	China	60/60	53.0	54.2	7.3	III 60.8% IV 39.2%	Acarbose 50mg Tid Gliquidone 30–60mg Tid	30 minutes each time, twice a day	CV12, LI11, LI4, ST36, SP9, SP6, ST40, SP10, SP8, LR3, BL30, BL23, BL43	5
Tang M 2022 (21)	China	34/34	49.0	66.2	2.6	/	Insulin Valsartan 80–160mg Qd	Moderate amount of time, once a day	BL23, BL20, GV4, ST36, SP6, SGX, CV6, CV4	12
Wang KX 2022 (22)	China	39/39	57.1	52.6	6.9	III 100%	Gliquidone 15–60mg Tid Candesartan 4–8mg Qd Simvastatin 5–10mg Qd	30 minutes each time, once a day	CV4, ST36, CV12, ST40, SP10, LR3	8
Wu YT 2008 (23)	China	20/22	51.6	45.2	1.8	/	Oral hypoglycemic drugs Benapride 10mg Qd	20 minutes each time, once a day	EX-B3, BL13, BL20, BL23, CV4, KI3, ST36, SP9, SP6	4
Yang XY 2013 (24)	China	27/27	55.0	/	/	/	Insulin Oral hypoglycemic drugs	30 minutes each time, once every two days	SP8, KI5, GV26, ST23, ST36, ST21, ST28, TE6, KI3, KI7, KI9, BL18, BL23, BL28, BL20, CV4, LI6, SP9, LR8, GB20	4
Zhu YP 2023 (25)	China	65/65	64.6	54.6	1.6	/	Insulin Oral hypoglycemic drugs Mecobalamin 0.5mg Tid	20 minutes each time, once every two days	ST44, ST43, SP2, SP3, LI2, LI3, SI2, SI3, PC8, PC7, ST36, ST37, ST39, ST40, SP8, SP6, SP9, LI4, ST25, CV12	4

*Baseline information such as gender, age, and disease duration were comparable between the experimental and control groups in each included study. SGX, acupoint named Shenguanxue."/" indicates that the study did not provide relevant data.

#### 3.1.3 Risk of bias assessment

**Figure 2:**
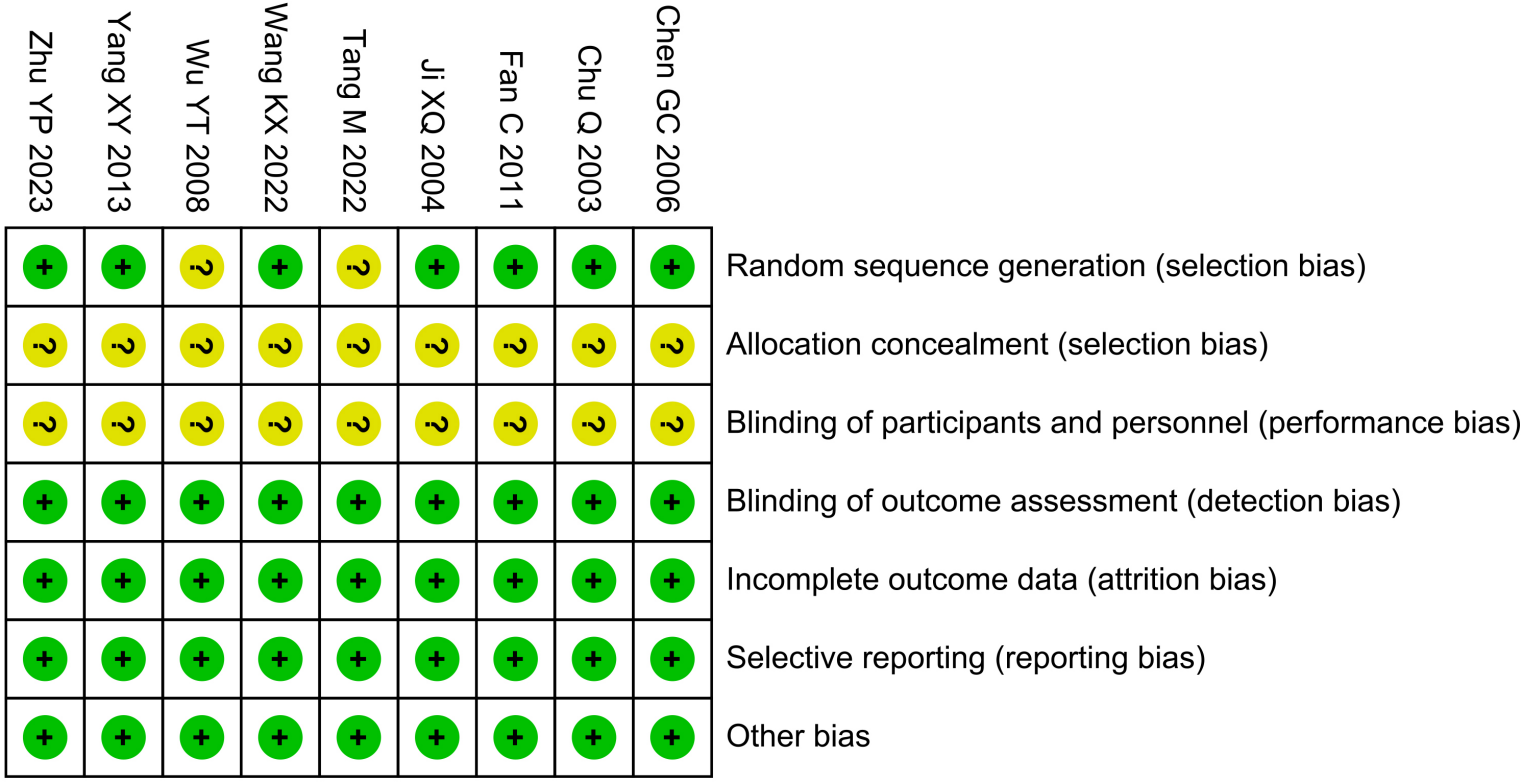
Risk of bias graph.

The risk of bias was unclear for the randomization method in two studies, the risk of bias for allocation concealment and blinding of interventions to patients and participants was unclear in nine studies, and the risk of bias was low in the remaining areas, as shown in **Figure 2**.

#### 3.1.4 Clinical effective rate

**Figure 3:**
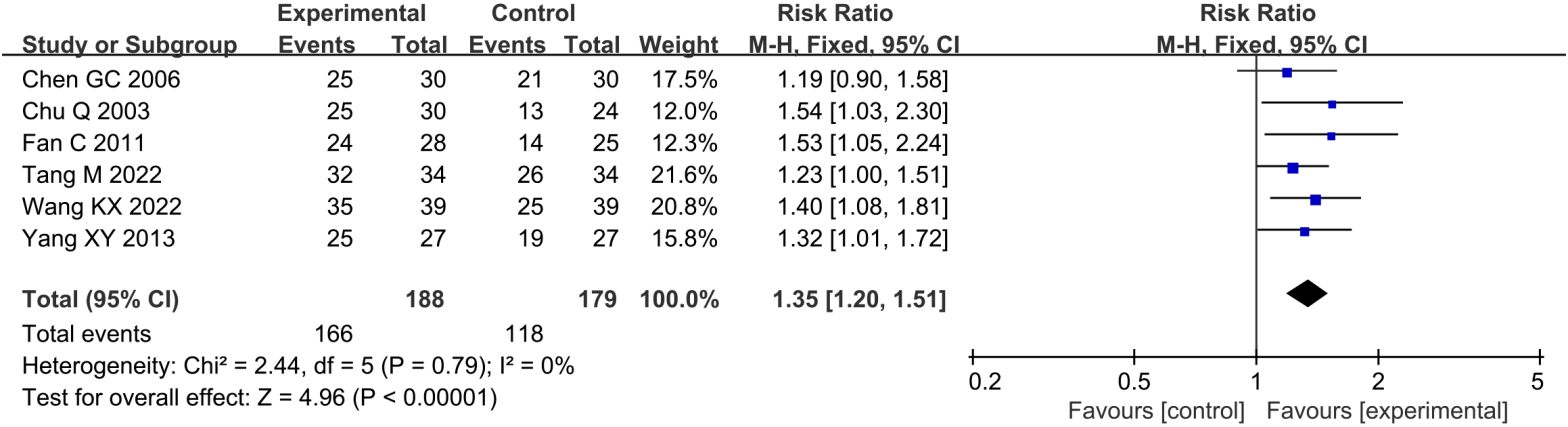
Meta analysis of clinical effective rate in acupuncture combined group vs conventional drug group in the treatment of DKD. DKD, diabetic kidney disease.

Compared with the conventional treatment group, the combined acupuncture group significantly increased the clinical effective rate by 35% (RR 1.35, 95% confidence interval [CI] 1.20 to 1.51, P < 0.00001), as shown in **Figure 3**.

#### 3.1.5 Renal function

**Figure 4:**
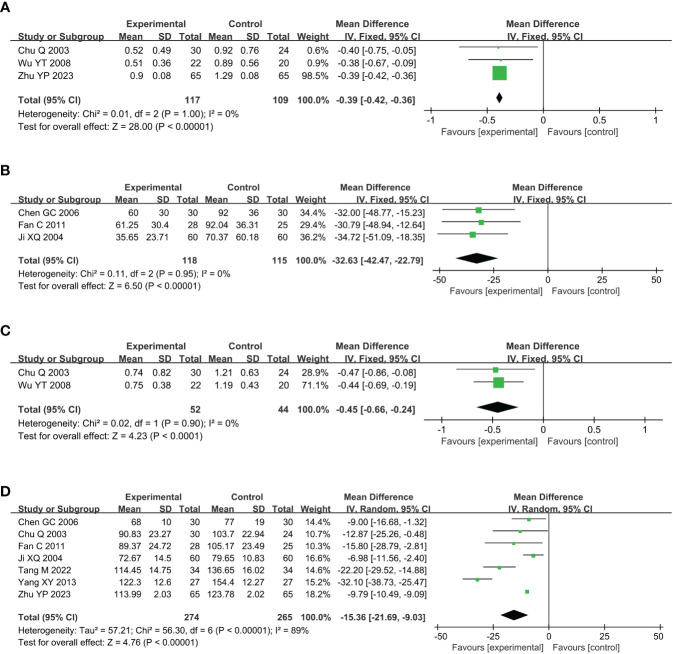
Meta analysis of renal function in acupuncture combined group vs conventional drug group in the treatment of DKD. **(A)** uALB, **(B)** umALB, **(C)** uβ2-MG; **(D)** SCR. DKD, diabetic kidney disease; uALB, urinary albumin; umALB, urinary microalbumin; uβ2-MG, urine β2 microglobulin; SCR, serum creatinine.

Compared with the conventional treatment group, the combined acupuncture group significantly decreased uALB by 0.39g (MD –0.39, 95% CI –0.42 to –0.36, P < 0.00001), umALB by 32.63mg (MD –32.63, 95% CI –42.47 to –22.79, P < 0.00001), uβ2-MG by 0.45μg/ml (MD –0.45, 95% CI –0.66 to –0.24, P < 0.0001), and SCR by 15.36μmol/L, as shown in **Figure 4**.

#### 3.1.6 Blood glucose

**Figure 5:**
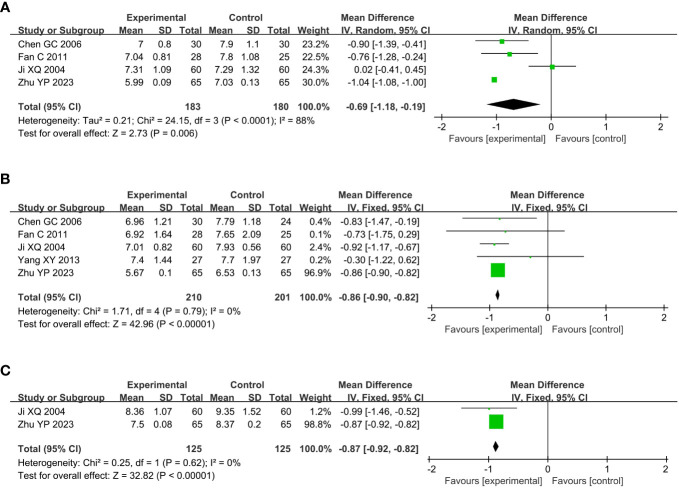
Meta analysis of blood glucose in acupuncture combined group vs conventional drug group in the treatment of DKD. **(A)** HbA1c, **(B)** FBG, **(C)** 2h PPG. DKD, diabetic kidney disease; HbA1c, glycated hemoglobin A1c; FBG, fasting blood glucose; 2h PPG, 2h postprandial plasma glucose.

Compared with the conventional treatment group, the combined acupuncture group significantly reduced HbA1c by 0.69% (MD –0.69, 95% CI –1.18 to –0.19, P = 0.006), FBG by 0.86mmol/L (MD –0.86, 95% CI –0.90 to –0.82, P < 0.00001) and 2h PPG by 0.87mmol/L (MD –0.87, 95% CI –0.92 to –0.82, P < 0.00001), as shown in **Figure 5**.

#### 3.1.7 Blood lipid

**Figure 6:**
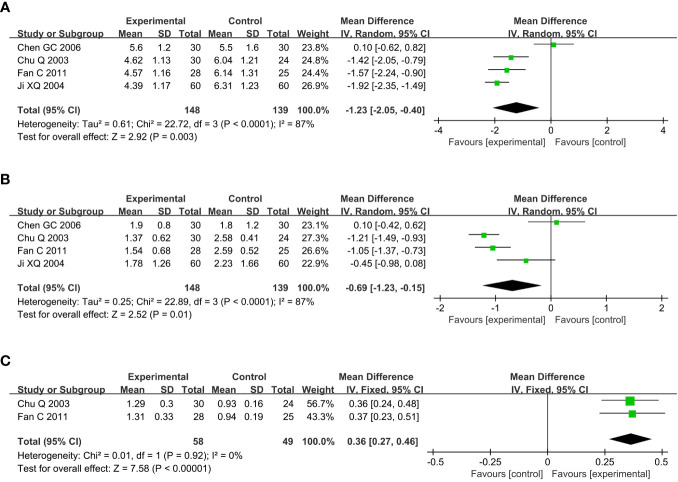
Meta analysis of blood lipid in acupuncture combined group vs conventional drug group in the treatment of DKD. **(A)** TC, **(B)** TG, **(C)** HDL-C. DKD, diabetic kidney disease; TC, total cholesterol; TG, triglyceride; HDL-C, high-density lipoprotein cholesterol.

Compared with the conventional treatment group, the combined acupuncture group significantly reduced TC by 1.23mmol/L (MD –1.23, 95% CI –2.05 to –0.40, P = 0.003) and TG by 0.69mmol/L (MD –0.69, 95% CI –1.23 to –0.15, P = 0.01), and increased HDL-C by 0.36mmol/L (MD 0.36, 95% CI 0.27 to 0.46, P < 0.00001), as shown in **Figure 6**.

#### 3.1.8 Safety endpoint

**Figure 7:**
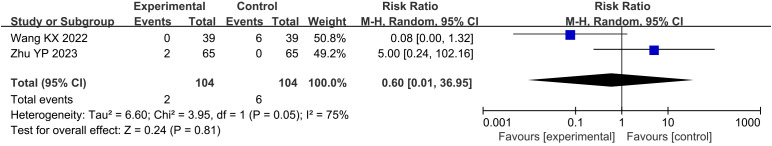
Meta analysis of adverse events in acupuncture combined group vs conventional drug group in the treatment of DKD. DKD, diabetic kidney disease.

Adverse events occurred in two patients in the combined acupuncture group, both of which were local hematomas caused by insufficient pressing time after needle withdrawal. There were six adverse events in the conventional treatment group, including three cases of vomiting and three cases of dyspepsia. There was no significant difference in adverse events between the combined acupuncture group and the conventional treatment group (RR 0.60, 95% CI 0.01 to 36.95, P = 0.81), as shown in **Figure 7**.

#### 3.1.9 Leave-one-out sensitivity analysis

The leave-one-out sensitivity analysis showed that the results of clinical effective rate, uALB, umALB, SCR, HbA1c, FBG, TC, and TG had low sensitivity and high confidence. Due to uβ2-MG, 2h PPG, HDL-C, and adverse events were only assessed in two of the included studies, their leave-one-out sensitivity analysis cannot be performed.

#### 3.1.10 Subgroup sensitivity analysis

The subgroup sensitivity analysis, with creatinine as the outcome, explored the effects of factors such as gender, age, and number of acupoints on the combined outcome. In terms of gender, acupuncture significantly reduced creatinine in the group of DKD with a male ratio of 41–50% (MD –14.27, 95% CI –23.23 to –5.30, P = 0.002), 51–60% (MD –14.18, 95% CI –22.47 to –5.89, P = 0.0008) and 61–70% (MD –22.20, 95% CI –29.52 to –14.88, P < 0.00001). In terms of age, acupuncture significantly reduced creatinine in the group of DKD with an average age of 41–50 years (MD –22.20, 95% CI –29.52 to –14.88, P < 0.00001), 51–60 years (MD –15.98, 95% CI –31.85 to –0.11, P = 0.048) and 61–70 years (MD –9.82, 95% CI –10.51 to –9.12, P < 0.00001). In terms of the number of acupoints, acupuncture significantly reduced creatinine in the group of DKD with 1–10 acupoints (MD –15.65, 95% CI –28.59 to –2.72, P = 0.02) and 10–20 acupoints (MD –15.28, 95% CI –23.31 to –7.26, P = 0.0002), as shown in **Table 2**.

**Table 2:** Subgroup sensitivity analysis of acupuncture treatment for diabetic kidney disease.

Subject	Subgroup	I^2^	MD (95% CI)	*p* value
Male ratio	41–50 %	0	–14.27 (–23.23, –5.30)	0.002
	51–60 %	93	–14.18 (–22.47, –5.89)	0.0008
	61–70 %	0	–22.20 (–29.52, –14.88)	<0.00001
Average age	41–50 years	0	–22.20 (–29.52, –14.88)	<0.00001
	51–60 years	95	–15.98 (–31.85, –0.11)	0.048
	61–70 years	0	–9.82 (–10.51, –9.12)	<0.00001
Number of acupoints	1–10 acupoints	83	–15.65 (–28.59, –2.72)	0.02
	11–20 acupoints	91	–15.28 (–23.31, –7.26)	0.0002

#### 3.1.11 Publication bias

**Figure 8:**
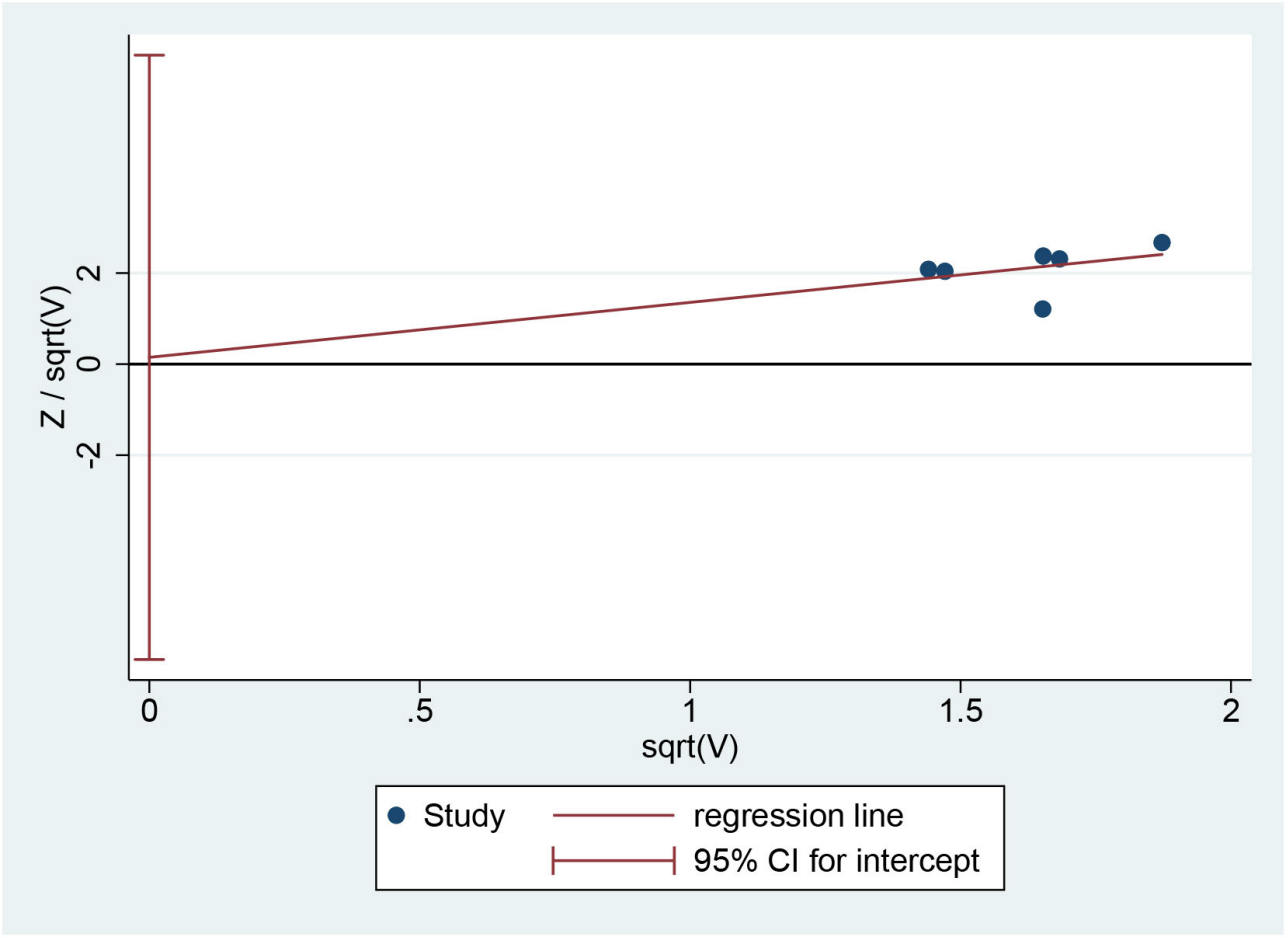
Harbord regression analysis for publication bias.

The Harbord regression of the clinical effective rate showed P = 0.953, suggesting no significant publication bias (**Figure 8**).

### 3.2 Data mining results

#### 3.2.1 Literature screening

A total of 789 relevant studies were obtained, and 51 clinical trials were included after stratified screening, containing 77 acupoints with a total frequency of 483.

#### 3.2.2 Frequency analysis

Frequency analysis was performed to obtain 20 common acupoints with a frequency greater than 20%, including Zusanli (ST36), Shenshu (BL23), Sanyinjiao (SP6), Taixi (KI3), Diji (SP8), Zhongwan (CV12), Yinlingquan(SP9), Pishu (BL20), Xuehai (SP10), Guanyuan (CV4), Tianshu (ST25), Taichong (LR3), Fenglong (ST40), Hegu (LI4), Weiwanxiashu (EX-B3), Baihuanshu (BL30), Quchi (LI11), Ganshu (BL18), Gaohuangshu (BL43), and Zhongji (CV3), as shown in **Table 3**.

**Table 3:** Common acupoints with a frequency greater than 20%.

Rank	Acupoint	Frequency (n/%)	Location
1	ST36	41 (80.39%)	On the lateral calf, 3 cun below ST35, on the line between ST35 and ST41.
2	BL23	40 (78.43%)	In the spinal region, 1.5 cun lateral to the lower border of the spinous process of the second lumbar vertebra.
3	SP6	33 (64.71%)	In the medial calf, 3 cun above themedial malleolus, on the posterior border of the medial aspect of tibia.
4	KI3	25 (49.02%)	In the ankle region, posterior to the medial malleolus, in the depression between the tip of the medial malleolus and tendo calcaneus.
5	SP8	22 (43.14%)	In the medial calf, 3 cun below SP9, on the posterior border of the medial aspect of tibia.
6	CV12	22 (43.14%)	In the epigastric region, 4 cun above the umbilicus, on the anterior midline.
7	SP9	19 (37.25%)	In the medial calf, in the depression of the lower border of the medial condyle of the tibia.
8	BL20	19 (37.25%)	In the spinal region, 1.5 cun lateral to the lower border of the spinous process of the eleventh thoracic vertebra.
9	SP10	18 (35.29%)	In the anterior femoral region, 2 cun above the medial end of patellar floor, on the bulge of the vastus medialis.
10	CV4	16 (31.37%)	In the lower abdomen, 3 cun below the umbilicus, on the anterior midline.
11	ST25	14 (27.45%)	On the abdomen, at the level of the umbilicus, 2 cun lateral to umbilicus.
12	LR3	14 (27.45%)	On the dorsum of the foot, between the first and second metatarsals, the anterior depression at the junction of the bases of the metatarsals.
13	ST40	13 (25.49%)	In the lateral calf, 8 cun sperior to the external malleolus, on the outer edge of the tibialis anterior muscle.
14	LI4	12 (23.53%)	On the dorsum of the hand, in the middle of he 2nd metacarpal bone on the radial side.
15	EX-B3	12 (23.53%)	In the spinal region, 1.5 cun lateral to the lower border of the spinous process of the eighth thoracic vertebra.
16	BL30	11 (21.57%)	In the sacral region, at the level of the fourth posterior sacral foramen, 1.5 cun lateral to the middle sacral crest.
17	LI11	11 (21.57%)	At the elbow, the midpoint of position of the line connecting LU5 and the lateral epicondyle of the humerus.
18	BL18	11 (21.57%)	In the spinal region, 1.5 cun lateral to the lower border of the spinous process of the ninth thoracic vertebra.
19	BL43	11 (21.57%)	In the spinal region, 3 cun lateral to the lower border of the spinous process of the fourth thoracic vertebra.
20	CV3	11 (21.57%)	In the lower abdomen, 4 cun below the umbilicus, on the anterior midline.

*ST35 is located in the anterior knee area, in the lateral depression of the patellar ligament. ST41 is located in the ankle area, in the central depression in front of the ankle joint. LU5 is located in the elbow area, on the transverse crease of the elbow, in the depression of the radial edge of the biceps tendon.

#### 3.2.3 Association rules analysis

**Figure 9:**
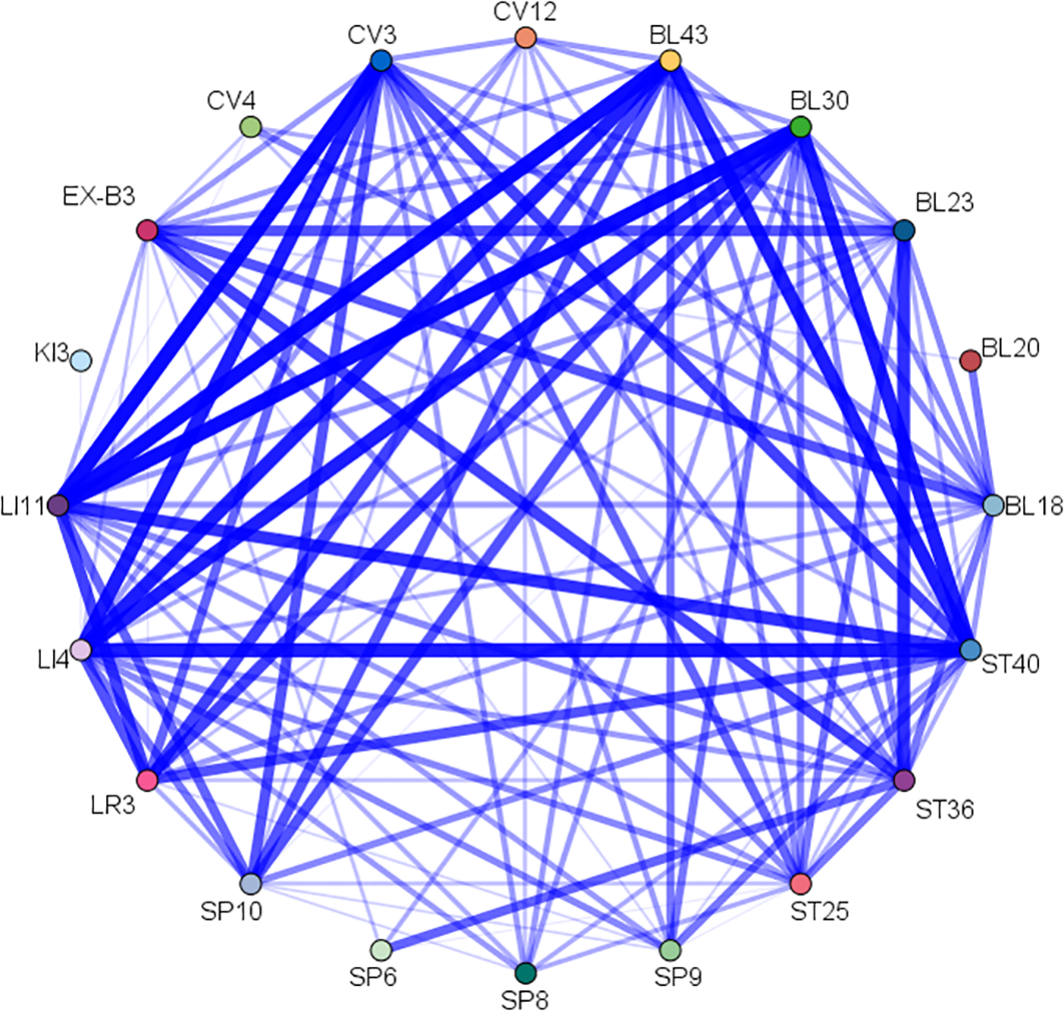
Network relationship for common acupoints.

Association rule analysis was used to explore core acupoint combinations consisting of common acupoints. Under the conditions of setting support ≥ 30%, confidence ≥ 100%, and lift ≥ 1.0, a total of 11 core acupoint combinations were obtained, as shown in **Table 4** They were formed by combining CV12, SP8, SP10, ST36, SP6, BL20, BL23, and SP9 with each other, indicating that these acupoints were the core acupoints for acupuncture in DKD. The network relationship diagram is shown in **Figure 9**.

**Table 4:** Association rules analysis of common acupoints.

Acupoint combination	Frequency/n	Support/%	Confidence/%	Lift
ST36, SP10	18	35.294	100.00	1.244
ST36, SP9	19	37.255	100.00	1.244
ST36, BL20, SP6	16	31.373	100.00	1.244
ST36, SP10, CV12	16	31.373	100.00	1.244
ST36, SP9, SP6	18	35.294	100.00	1.244
ST36, SP9, BL23	18	35.294	100.00	1.244
ST36, CV12, BL23	17	33.333	100.00	1.244
ST36, SP8, SP6	16	31.373	100.00	1.244
ST36, SP8, BL23	17	33.333	100.00	1.244
ST36, SP9, SP6, BL23	17	33.333	100.00	1.244
ST36, CV12, SP8, BL23	16	31.373	100.00	1.244

#### 3.2.4 Factor analysis

**Figure 10:**
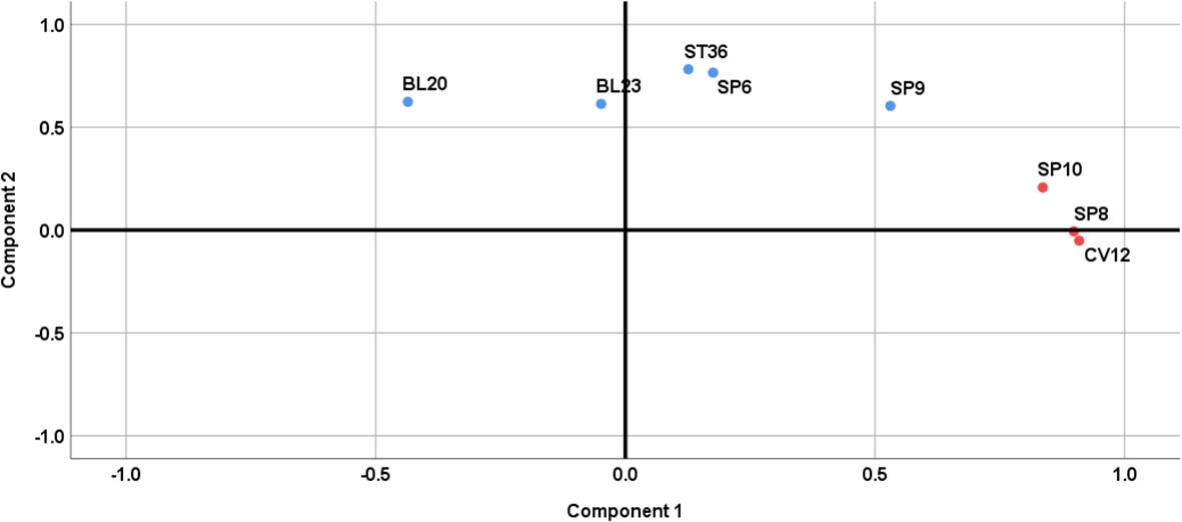
Factor analysis for core acupoints.

Factor analysis was employed to explore the combinatorial relationship between the core acupoints, as shown in **Figure 10**. A total of two common factors were obtained, and the cumulative variance contribution was 65.30%. Common factor 1 consisted of CV12, SP8, and SP10, which accounted for 35.66% of the total variance. Common factor 2 consisted of ST36, SP6, BL20, BL23, and SP9, accounting for 29.64% of the total variance.

## 4 Discussion

### 4.1 Research background and significance

DKD is a major cause of end-stage renal disease (26). Patients with DKD have a higher mortality rate compared to diabetic patients without renal disease (27), and their prevalence has shown a gradual increase (28). Currently, there are no specific drugs for DKD, and strict control of blood glucose and blood pressure remains the primary strategy in treating DKD (29). Although these therapeutic strategies have delayed the progression of DKD to some extent, some patients still eventually develop end-stage renal disease (30). Acupuncture has been reported to have unique efficacy and a favorable safety profile in treating diabetes and its complications (31). In 1983, Li RC et al. (32) confirmed for the first time through animal experiments that acupuncture promotes insulin secretion and improves glucose tolerance, which has since opened the study of acupuncture for diabetes and its complications. Subsequent researchers have found that acupuncture not only reduces glycemia (33) but also has a combined effect of regulating lipids and hemodynamics (34, 35). In 2001, Zhang YL et al. (36) first reported the efficacy of acupuncture in the treatment of DKD, and they found that acupuncture could reduce 24-hour urine protein quantification and SCR in patients with DKD. Since then, there has been increasing evidence of the role of acupuncture in improving the prognosis of DKD (37). Nevertheless, the specific benefits and acupoint selection schemes of acupuncture for DKD are still controversial. Therefore, this study aimed to review and collect clinical trials of acupuncture for DKD, elaborate on the specific benefits of acupuncture for DKD through meta-analysis, and then explore the acupoint selection of acupuncture for DKD through data mining.

### 4.2 Evaluation of efficacy

The study results showed that the clinical effective rate of the combined acupuncture group was significantly higher than that of the conventional treatment group, suggesting that acupuncture could effectively reduce the symptoms and signs of DKD patients. In terms of renal function, compared with the conventional treatment group, the combined acupuncture group significantly reduced 15.36μmol/L SCR, 0.39g uALB, 32.63mg umALB and 0.45μg/ml uβ2-MG. SCR is an essential indicator that reflect the function of the kidney to remove metabolic waste (38). uβ2-MG is often used to evaluate renal tubular function (39). ALB reflects glomerular injury and glomerular permeability to macromolecules (40). mALB is an indicator of renal and systemic endothelial dysfunction (41). This evidence suggests that acupuncture effectively improves renal function in patients with DKD, thereby bettering the prognosis of DKD.

On the glycemic-related endpoints, the combined acupuncture group significantly reduced 0.69% HbA1c, 0.86 mmol/L FBG, and 0.87 mmol/L 2h PPG compared with the conventional treatment group, implying that acupuncture has a glycemic lowering effect. Actually, hyperglycemia, hypertension, obesity, and dyslipidemia are major risk factors for the development and progression of DKD (42). It has been reported that chronic persistent hyperglycemia can cause abnormal glucose and lipid metabolism, hemodynamic changes, and oxidative stress in the body, which can lead to renal dysfunction and DKD (43, 44). Studies have shown that the incidence of diabetic complications is directly related to blood glucose levels (45), and the risk of microvascular complications increases by 40% for every 1% increase in HbA1c levels (46). The rate of renal impairment in patients with DKD is also controlled by blood glucose (47). Nosadini R et al. (48) noted that when HbA1c is consistently greater than 7.5% and postprandial glucose is more significant than 200 mg/dl, the risk of a rapid decline in glomerular function is significantly increased. On lipid-related endpoints, compared with the conventional treatment group, the combined acupuncture group significantly reduced 1.23 mmol/L TC and 0.69 mmol/L TG and significantly increased 0.36 mmol/L HDL-C, suggesting that acupuncture has an additional benefit in lipid modulation. In addition, considering that cardiovascular events are one of the main causes of death in DKD patients, regulating blood lipids can help improve the cardiovascular prognosis of DKD patients (27).

### 4.3 Evaluation of safety

On the safety endpoints, adverse events in the combined acupuncture group were not significantly different from those in the conventional treatment group, suggesting that acupuncture has a good safety profile and does not additionally increase the risk of adverse events.

Although meta-analysis showed that acupuncture was a safe treatment for DKD, it is still essential to be aware of potential adverse events such as hematoma, pain, infection, and needle-sickness that may be associated with acupuncture (49, 50). (1) Hematoma: Hematoma is one of the most prevalent untoward incidents following needle insertion. In this study, both adverse events observed in the acupuncture group were hematoma, typically arising from inadequate post-needle removal compression time. Applying gentle pressure with a cotton swab for approximately 30 seconds upon needle extraction effectively prevents hematoma formation. Hematomas formed by minimal bleeding generally resolve independently without requiring specific interventions. In cases where excessive subcutaneous bleeding induces intense pain, initial hemostasis through cold compression is recommended, followed by warm compression 24 hours later to facilitate hematoma absorption. (2) Pain: As an invasive therapeutic modality, acupuncture may induce localized pain at the needling site. Clinical practitioners should rigorously control needle insertion angles, depths, and stimulation levels in adherence to guidelines to alleviate the patient’s pain burden. Additionally, severe pain is commonly attributable to hematoma formation; hence, averting hematoma development effectively reduces the incidence of pain. (3) Infection: Infections typically arise from inadequate disinfection during operation. Given the slower wound healing in diabetic patients compared to the general population, infections are more prone to occur in patient with DKD. Strict adherence to standardized disinfection protocols significantly mitigates the risk of infection. (4) Needle-sickness: Needle-sickness refers to symptoms such as dizziness, palpitations, nausea, and sweating during the needling process. The precise mechanism of it remains not entirely elucidated, but it is generally associated with factors such as psychological tension, hunger, improper positioning, and excessive stimulation. Pre-needling education and effective doctor-patient communication contribute to reducing occurrences of needle-sickness. In the event of needle-sickness, immediate cessation of the procedure is necessary, allowing the patient to recover gradually.

### 4.4 Mechanisms of acupuncture for DKD

Currently, studies on the mechanisms of acupuncture in the treatment of DKD have focused on podocytes. Podocytes are terminally differentiated and highly specialized cells whose foot processes can attach to the glomerular basement membrane and connect to each other through the slit diaphragm (SD) (51). Podocyte injury is one of the earliest pathological changes in DKD (52), which mainly manifests as loss of foot processes and destruction of SD (53). Studies have shown that acupuncture can protect podocytes from damage by restoring SD density, up-regulating the expression of SD proteins (nephrin and CD2 associated protein) and the apical membrane protein podocalyxin, and downregulating the cytoskeletal intermediate filament protein desmin (51). Acupuncture can also up-regulate the expression of podocyte mucin, thereby exerting a protective effect on the top surface of podocytes (51). Liu L et al. (54) found that acupuncture can improve the podocyte damage of rats with DKD and alleviate pathological changes such as glomerular basement membrane thickening by up-regulating the expression of podocalyxin, CD2 associated protein, and nephrin and down-regulating the expression of desmin, thereby delaying the progression of DKD.

Oxidative stress and inflammation are also closely related to podocyte damage (55). Wang M et al. (56) proposed that acupuncture can regulate the oxidative stress state of patients with DKD by down-regulating the contents of malondialdehyde, protein carbonyl, and 8-hydroxyguanine in plasma and up-regulating the activity of superoxide dismutase in patients with DKD. Wang KX et al. (22) found that acupuncture can reduce oxidative stress by up-regulating the expression of forkhead box protein O1 and peroxisome proliIerators-activated receptor γ coactivator lalpha, thereby protecting the kidneys. Zhang J et al. (57) reported that acupuncture can reduce renal inflammatory injury by inhibiting Nod-like receptor thermal protein domain associated protein 3/nuclear factor-kappa B pathway. In addition, acupuncture can also alleviate kidney injury in rats with DKD by promoting renal cell autophagy (58).

### 4.5 Data mining of acupuncture scheme

The frequency analysis yielded 20 common acupoints for acupuncture in DKD. However, limited by the support and confidence level, common acupoints such as KI3, CV4, ST25, LR3, ST40, LI4, EX-B3, BL30, LI11, BL18, BL43, and CV3 were excluded from the association rule analysis. Although it contained commonly used acupoints for the treatment of diabetes and its complications, such as LI4 and LI11, the association rule analysis showed that these acupoints were underutilized or lacked close association with other acupoints in the database constructed by the included studies. The association rule analysis yielded core acupoint combinations were composed of CV12, SP8, SP10, ST36, SP6, BL20, BL23, and SP9, suggesting that these acupoints were the core acupoints for acupuncture in DKD. The needling methods and location plan for core acupoints are shown in **Table 5**.

**Table 5:** Acupuncture strategy for diabetic kidney disease based on data mining.

Acupoint	Needling Methods	Location Plan
ST36	Perpendicular insertion 1–2 cun (about 2–4 cm)	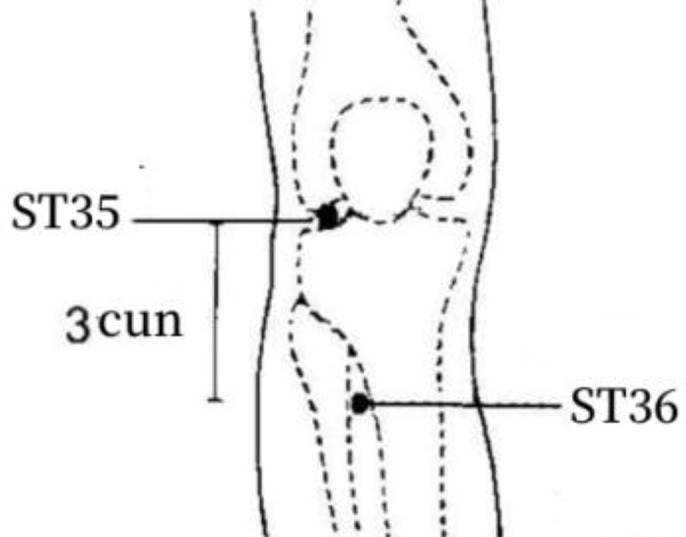
BL23	Perpendicular or 45-degree angle towards the spine insertion 0.5–1 cun (about 1–2 cm)	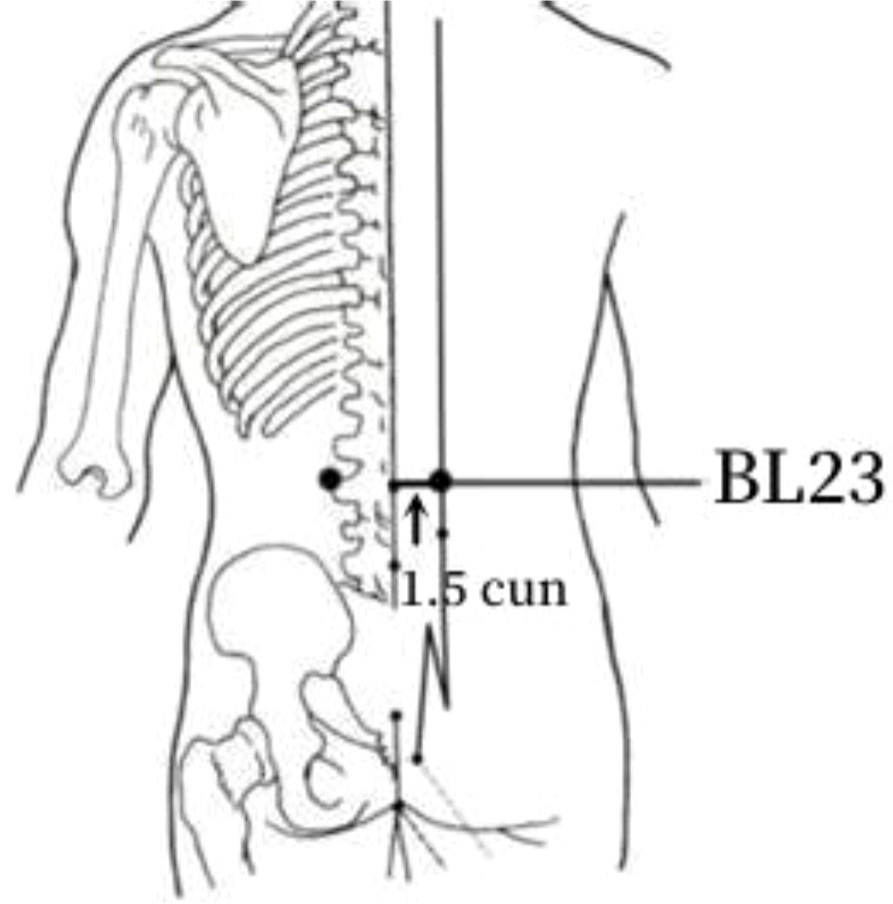
SP6	Perpendicular insertion 1–1.5 cun (about 2–3 cm)	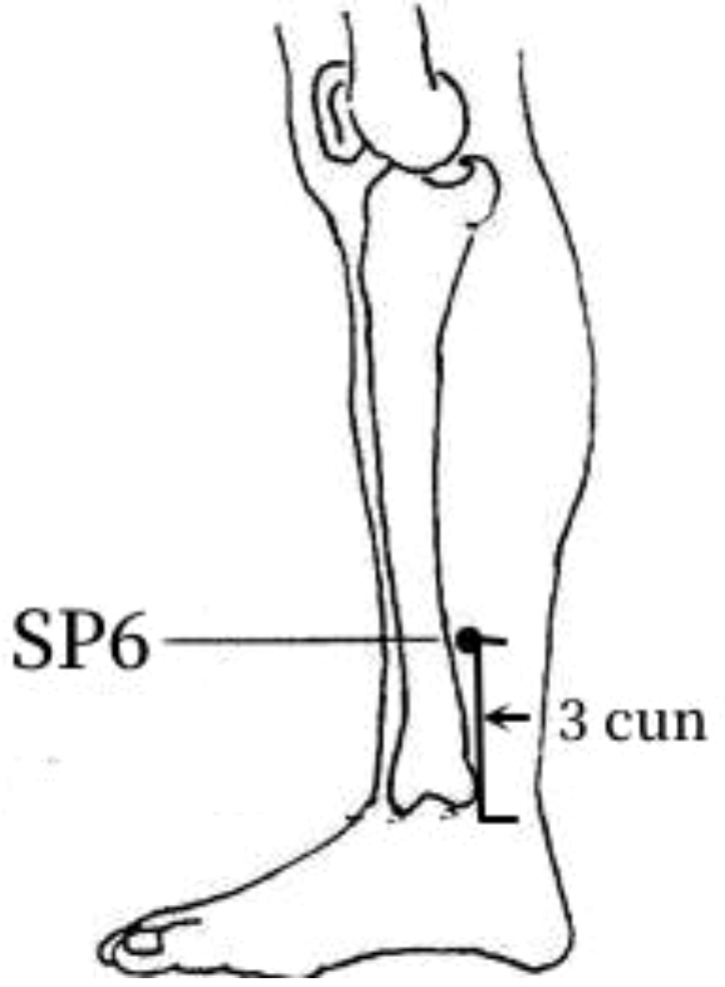
SP8	Perpendicular insertion 1–1.5 cun (about 2–3 cm)	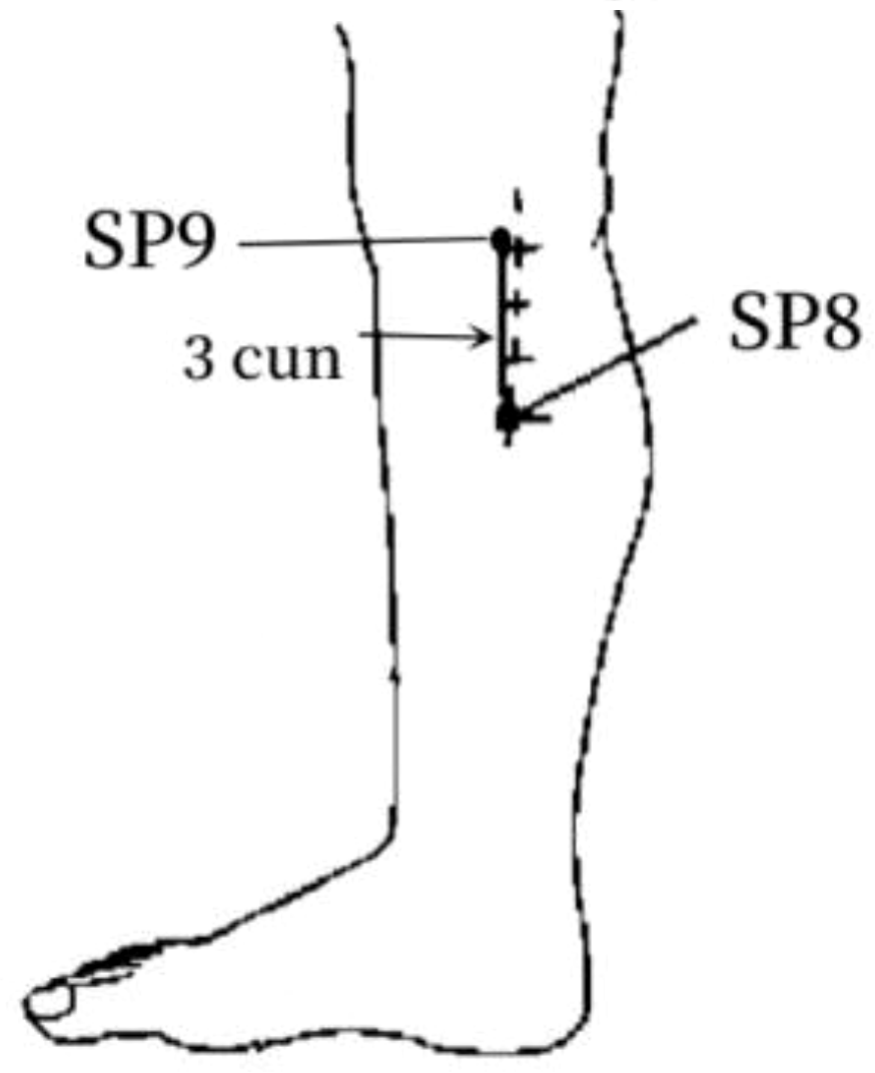
CV12	Perpendicular insertion 1–1.5 cun (about 2–3 cm), with caution in pregnant women and after urination.	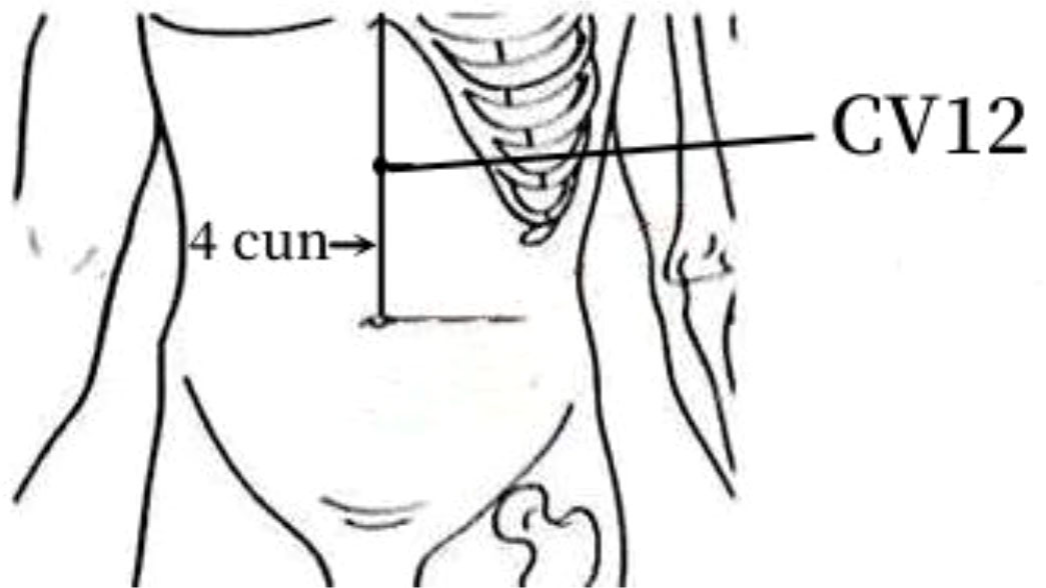
SP9	Perpendicular insertion 1–2 cun (about 2–4 cm)	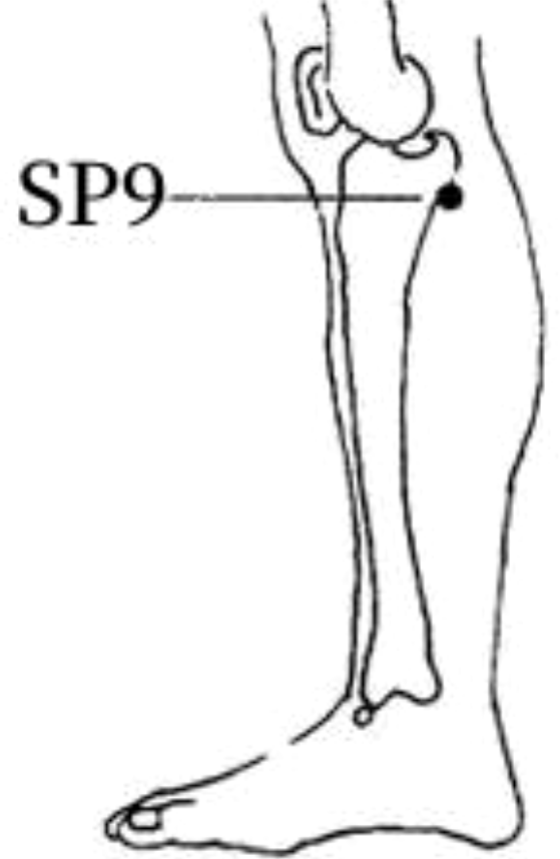
BL20	45-degree angle towards the spine insertion 0.5–0.8 cun (about 1–2 cm)	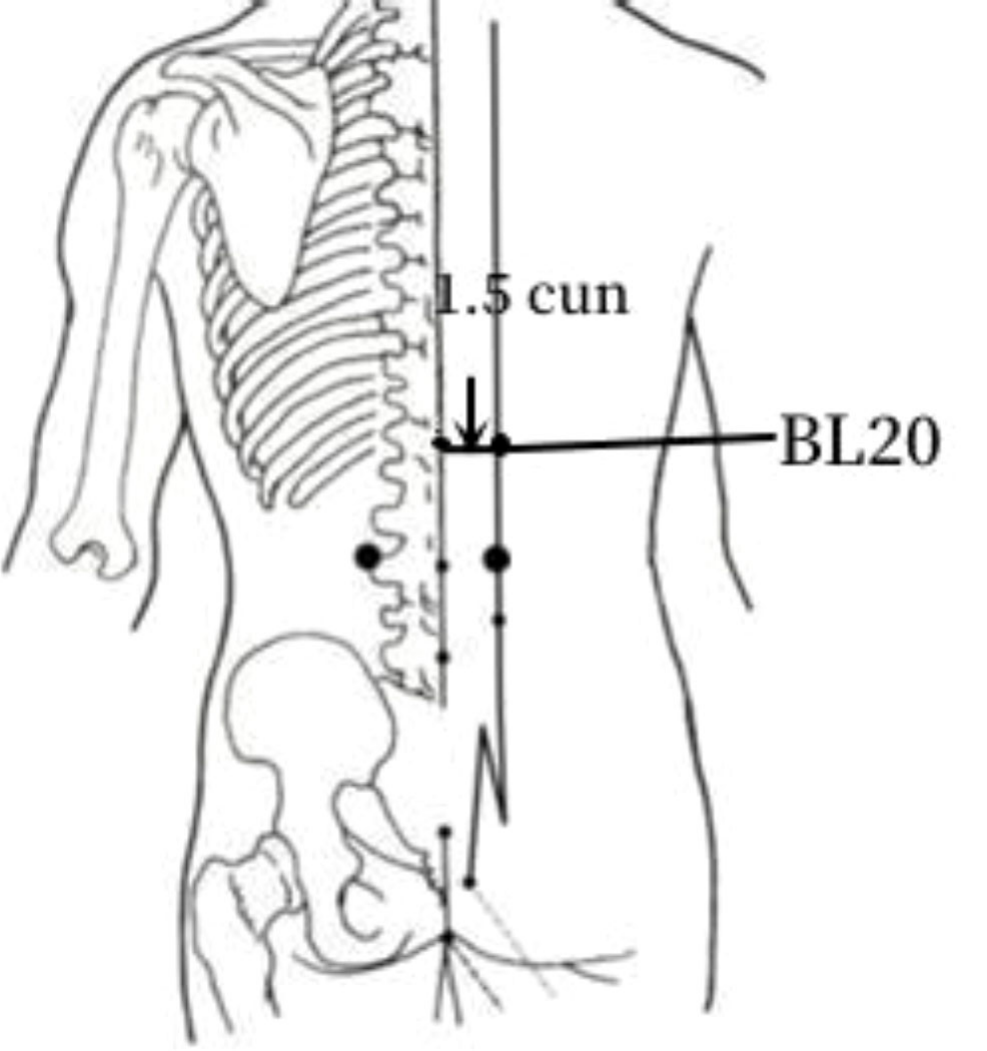
SP10	Perpendicular insertion 1–1.5 cun (about 2–3 cm)	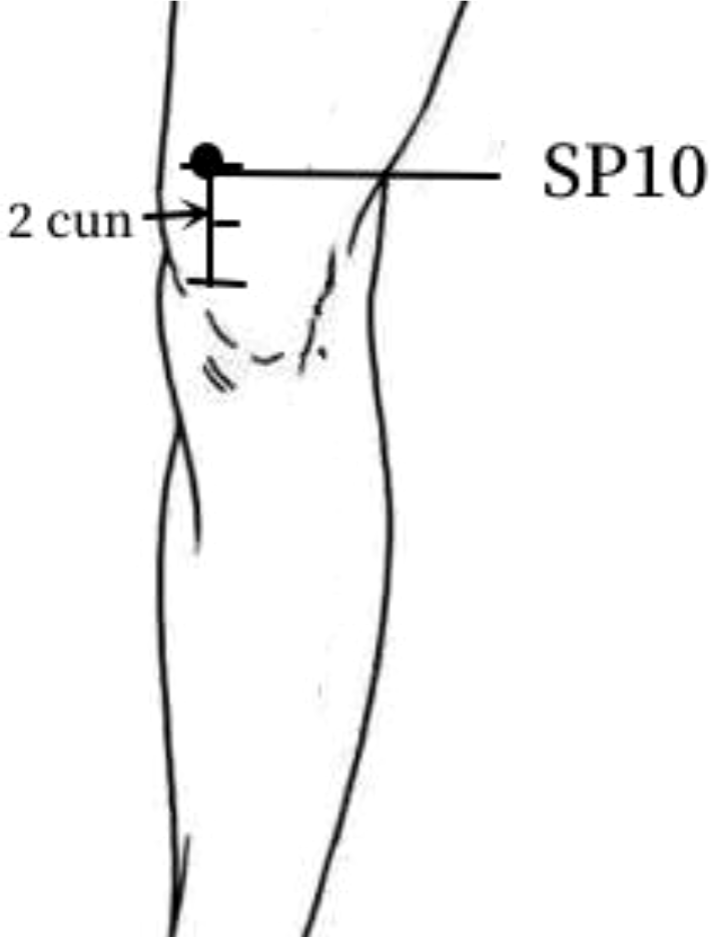

Traditional Chinese medical theory posits that the pathogenesis of DKD is intricate, encompassing both spleen-kidney deficiency as well as phlegm and blood stasis obstruction (59). Given the selection of specific acupoints was based on the Chinese medical pathogenesis, the intricate pathogenesis of DKD dictate the requirement for multiple acupoint combinations in its treatment. In this study, factor analysis categorized the core acupoints into two groups. The first group comprised CV12, SP8, and SP10, primarily functioning to tonify the kidneys and dissipate blood stasis. The second group consisted of ST36, SP6, BL20, BL23, and SP9, primarily acting to tonify the spleen and kidney, and dissipate phlegm. These two sets of acupoints complement each other, collectively exerting the therapeutic effects of tonifying the spleen and kidneys as well as dissipating phlegm and blood stasis, aligning with the Chinese medical pathogenesis of DKD. Hence, acupuncturists must comprehend the significance of these core acupoints and their groupings in the treatment of DKD and fully apply them in clinical practice.

### 4.6 Limitation and outlook

Although the study followed the PRISMA guidelines, there are still some limitations. First, only 659 samples were included in the meta-analysis part of the study, and only 51 programs were included in the data mining part, which may lead to a decrease in the accuracy of the results. Second, Wang KX et al. (22) limited the age to 30–70 years and only included patients with Mogensen stage III, while Chu Q et al. (18) subjectively excluded patients with blood glucose >14 mmol/L. The limitations of these inclusion criteria may reduce the generalizability of the meta-analysis results. Third, the research centers of the nine clinical trials were all in China, and the study subjects were mainly of Chinese descent. This means that the results of this study were mainly used to evaluate the effects of acupuncture in Chinese patients with DKD, and it is not yet clear how the therapy works in Europeans, Americans, and Africans. Fourth, the course of treatment included in the study was between four and 12 weeks, so the meta-analysis results mainly reflected the short-term efficacy of acupuncture in treating DKD, and there was a lack of long-term follow-up data.

Given the limitations of existing research, we expect the future studies will continue to improve: (1) The acupuncture strategy obtained in this study can be used in clinical trials to verify its efficacy and safety in treating DKD. (2) By facilitating the development of multicenter stratified studies, further explore the efficacy of acupuncture in treating patients with DKD of varying age, stage, baseline glucose level, and baseline weight. (3) Research centers can be established in European, American, and African countries to further evaluate the specific benefits of acupuncture in different ethnic populations. (4) Researchers can further evaluate the long-term efficacy of acupuncture in treating DKD by extending the follow-up period to half a year to one year.

## 5 Conclusion

Acupuncture improved clinical symptoms, renal function indices such as uALB, umALB, uβ2-MG, and SCR, as well as blood glucose and blood lipid in patients with DKD, and has a favorable safety profile. CV12, SP8, SP10, ST36, SP6, BL20, BL23, and SP9 are the core acupoints for acupuncture in DKD, and this program is expected to become a supplementary treatment for DKD.

## Data availability statement

The original contributions presented in the study are included in the article/supplementary material. Further inquiries can be directed to the corresponding author.

## Author contributions

YFY: Conceptualization, Supervision, Writing – original draft. GH: Methodology, Supervision, Writing – original draft. XY: Formal analysis, Methodology, Writing – original draft. YMY: Data curation, Formal analysis, Writing – original draft. KT: Data curation, Formal analysis, Writing – original draft. RY: Writing – review & editing.
